# Fluorescence Lifetime Imaging Combined with Conventional Intravascular Ultrasound for Enhanced Assessment of Atherosclerotic Plaques: an Ex Vivo Study in Human Coronary Arteries

**DOI:** 10.1007/s12265-015-9627-3

**Published:** 2015-05-01

**Authors:** Hussain Fatakdawala, Dimitris Gorpas, John W. Bishop, Julien Bec, Dinglong Ma, Jeffrey A. Southard, Kenneth B. Margulies, Laura Marcu

**Affiliations:** Department of Biomedical Engineering, University of California Davis, 451 E. Health Sciences Drive, Davis, CA 95616 USA; Department of Pathology & Laboratory Medicine, University of California Davis Medical Center, Davis, CA USA; Division of Cardiovascular Medicine, University of California Davis Medical Center, Davis, CA USA; Cardiovascular Institute, Perelman School of Medicine, University of Pennsylvania, Philadelphia, PA USA

**Keywords:** Atherosclerosis, Cardiovascular diseases, Intravascular imaging, Fluorescence lifetime imaging, Intravascular ultrasound

## Abstract

This study evaluates the ability of label-free fluorescence lifetime imaging (FLIm) to complement intravascular ultrasound (IVUS) for concurrent visualization of human coronary vessel composition, structure, and pathology. Co-registered FLIm and IVUS data from 16 coronary segments were correlated to eight distinct pathological features including thin-cap fibroatheroma (TCFA). The sensitivity, specificity, and positive predictive value for combined FLIm-IVUS (89, 99, 89 %) were better than FLIm (70, 98, 88 %) and IVUS (45, 94, 62 %) alone in distinguishing between pathologies. FLIm can assess compositional changes in luminal surface through variations in fluorescence lifetime values (<3.5 ns for lipid-rich areas; >4 ns for collagen-rich areas) enabling detection of macrophages in fibrous caps (sensitivity, 86 %) and distinguishing between relatively stable thick-cap fibroatheromas and rupture-prone TCFA (sensitivity, 80 %) amongst other features. Current results demonstrate the potential of FLIm-IVUS as a new intravascular method for improved evaluation of plaques that may subsequently aid in guiding coronary intervention.

**Electronic supplementary material:**

The online version of this article (doi:10.1007/s12265-015-9627-3) contains supplementary material, which is available to authorized users.

## Introduction

Majority of sudden acute cardiovascular events are caused due to rupture or erosion of vulnerable plaques [1]. The identification of these plaques can aid in the advancement of interventional techniques and pharmacological therapies to reduce such events. Since angiography provides limited information about the nature of the plaque occluding a vessel, percutaneous coronary interventions (PCIs) assisted with imaging techniques have been realized as an important tool for studying plaque progression and pathology in animals in vivo and in patients.

Optical coherence tomography (OCT) and intravascular ultrasound (IVUS) are the two most common intravascular imaging techniques used in the catheterization laboratory [2] with near-infrared spectroscopy (NIRS) recently introduced into clinical practice as well [3]. IVUS is not able to detect rupture-prone thin-cap fibroatheromas (TCFAs) due to limited spatial resolution (>100 μm). Conversely, OCT has a spatial resolution of 10–20 μm and can detect thin caps [4] as well as assess inflammation [5] but has limited imaging penetration depth and cannot reliably determine the size of deep plaque cores or outward remodeling, an important indicator of plaque vulnerability. NIRS has the ability to detect lipid cores but has limited sensitivity for other proteins of interest such as collagen and elastin. A comparison of these techniques is illustrated in Table T1 in the supplemental material. Since each modality by itself is limited in one way or another, multimodal imaging where two or more modalities complement one another to allow simultaneous assessment of plaque structure and composition would be extremely beneficial.

The overall objective of this study is to demonstrate the following: (1) imaging results from diseased human coronary vessels using a novel label-free bimodal intravascular scanning technique that combines fluorescence lifetime imaging (FLIm) and IVUS and (2) the ability of FLIm to complement conventional IVUS for improved human coronary plaque assessment via quantification of plaque composition. Previous work has shown the potential of endogenous fluorescence lifetime measurements for atherosclerotic plaque characterization in open arteries [6, 7]. This paper reports the first results from human coronary arteries interrogated with such a bimodal rotational catheter that serves as an important feasibility step prior to studying plaques in an in vivo animal model as well as future human studies. We explore the ability of FLIm in detecting biochemical features (e.g., infiltration of macrophages in fibrous caps) and distinguishing between rupture-prone thin-cap fibroatheromas (TCFAs) from relatively stable thick-cap fibroatheromas (ThCFAs) that can complement morphological features derived from conventional IVUS.

## Methods

### Specimens

The human left anterior descending (LAD) coronary artery samples used in these studies were obtained from 16 different patients from the University of Pennsylvania with approval from the institutional review board (additional information in the supplemental material). Samples were chilled in isopentane and rapidly frozen in liquid nitrogen and shipped overnight and stored at −80 °C.

### FLIm-IVUS Imaging Studies

The FLIm-IVUS bimodal imaging system and rotational catheter (5-Fr shaft and 3-Fr imaging section) used in this study have been previously described in detail [8] and can perform sequential IVUS and FLIm helical scans. Details of the FLIm subsystem are described in the supplemental material. Briefly, it is composed of a 300-μm core ultraviolet (UV)-grade silica/silica side-viewing optical fiber (Polymicro, Phoenix, AZ) rotating at 1200 rpm delivering UV light (Fianium, 355 nm, 80-ps pulse width) with a repetition rate of 10 KHz and an energy of ~100 nJ/pulse. The IVUS subsystem (Atlantis SR Pro with iLab, Boston Scientific, MA, USA) is composed of a single element transducer (40 MHz) driven at a rotation speed of 1800 rpm with data sampled at 200 MHz. Both FLIm and IVUS imaging were performed with a pullback speed of 2 mm/s sequentially. FLIm had an axial/angular resolution of 160 μm/14° while IVUS had an axial/lateral resolution of 100 μm/200 μm. A 20-mm pullback took 10 s for each modality. Data acquisition, saving, and processing took 6 min in total [8]. FLIm-IVUS co-registration was performed offline.

The specimen was imaged in a custom-built holder placed in a phosphate-buffered saline bath maintained at 37 °C. The specimen was fastened on to luers that presented a notch painted with fluorescent ink so that they were visible on both FLIm and IVUS images to allow axial and angular co-registration of both modalities (Fig. S1 in the supplemental material). Additionally, a guide wire (0.014″) was also placed in the lumen that was visible on both modalities to aid in angular registration. The guide wire was immobilized by fastening it in place using the screw end of the luer receiver (Fig. S1c in the supplemental material). It must be noted that FLIm-IVUS imaging was performed in saline and not blood. In vivo FLIm imaging will require clearance of blood as is done during OCT imaging. Tissue processing for acquiring co-registered histology is detailed in the supplemental material. A total of 87 histological sections were cut at 2-mm intervals from all specimens. For each cut, FLIm-IVUS data were averaged from the corresponding registered frame and the two adjacent frames. All histology slides were digitized at ×20 magnification (Aperio ScanScope, Aperio Technologies) for morphometric analysis.

### Pathological Features and Morphometric Analysis

Eight different pathological features were characterized in this study (Table 1) following definitions published by Virmani et al. [9]. Representative histology images for various features are shown in Fig. S2 in the supplemental material. On the digitized histology images, these features were identified by a pathologist as regions of interest (ROIs). Characteristic descriptors such as the amount of collagen, elastin, lipids/cholesterol, calcium, smooth muscle cells, and macrophages/lymphocytes were observed on pathology that allowed assigning a given ROI to one of the eight features. Significant macrophage/lymphocyte infiltration in tissue was determined by identifying the presence of >10 % clusters of differentiation (CD) 68+/CD45+ staining area in a given ROI. ROI selection and measurement of fibrous cap thickness were performed using a commercial software (Aperio ImageScope, Aperio Technologies). Corresponding co-registered imaging data were extracted by defining the same ROI on IVUS images. FLIm data were mapped onto the IVUS lumen [10] and attributed to the corresponding ROI. The ROIs on IVUS were scaled up in size proportional to the change in the scale bars between IVUS and histology when both images were co-registered. This change in ROI size between IVUS and histology is due to shrinkage in tissue after histological processing.

**Table 1:** Summary of pathological features observed in this study

Abbreviation	Pathological feature	Number of ROIs	Description
DIT	Diffuse intimal thickening	69	Mild intimal thickening comprised of loose collagen fibers and smooth muscle cells with intact elastic lamina rich in elastin fibers.
PIT	Pathological intimal thickening	32	Substantial intimal thickening with smooth muscle cells and high content of loose as well as densely packed collagen fibers. Sparse macrophages/lymphocytes (<1 % CD68+ and CD45+ stain) and lipids may be seen as well.
ThCFA	Thick-cap (>65 μm) fibroatheroma	14	Fibroatheroma with a thick cap (>65 μm) and underlying necrotic core. Cap is rich in collagen and smooth muscle cells and may have sparse macrophages/lymphocytes (<1 % CD68+ and CD45+ stain). Core may be lipid/cholesterol rich, cellular with macrophages and lymphocytes (>10 % CD68+ and CD45+ stain), and smooth muscle cells with presence of collagen. Core may also have calcium deposits.
ThCFAM	Thick-cap (>65 μm) fibroatheroma with macrophage/lymphocyte infiltration in cap	7	Similar to ThCFA but the cap has prominent infiltration of macrophage/lymphocytes (>10 % CD68+ and CD45+ stain).
TCFA	Thin-cap (<65 μm) fibroatheroma	10	Fibroatheroma with a thin cap (<65 μm) rich in macrophages/lymphocytes (>10 % CD68+ and CD45+ stain) and sparse smooth muscle cells. A prominent core is present that is lipid/cholesterol rich and cellular with macrophages and lymphocytes. Core may also show intraplaque hemorrhage or presence of calcium deposits.
FC	Fibrocalcific plaque	36	Fibroatheroma with cap that is predominantly calcified with high collagen content. May have a small core with calcium as well (nodules).
FT	Fibrotic tissue	12	Tissue regions in a stable fibrous cap or core that is predominantly composed of densely packed mature collagen (acellular).
LFA	Lipid-rich core of fibroatheroma (thick cap)	4	Large predominantly lipid-rich core beneath a thick fibrous cap. Contains large amount of extracellular lipid and cholesterol. May be rich in macrophages and lymphocytes (>10 % CD68+ and CD45+ stain).

*ROI* region of interest

### Imaging Parameters

A total of five IVUS-derived parameters and 42 FLIm-derived parameters were studied. The IVUS parameters included log-compressed ultrasound intensity (UI), integrated backscatter (IB) values, energy norm (*E*), intimal thickness (IT), and radial distance (*R*) of the ROI from the center of the IVUS transducer. The computation of these parameters have been previously reported [11] and are described in detail in the supplemental material. UI, IB, and *E* represent the amount of sound backscattered from tissue and may allow differentiating between different tissue types.

FLIm data includes fluorescence measurements from three sub-bands with central wavelength/bandwidth of 390/40 nm (channel 1 (CH1)), 452/45 nm (channel 2 (CH2)), and 542/50 nm (channel 3 (CH3)), respectively. CH1 encompasses fluorescence predominantly from collagen, CH2 from elastin and NADH, and collagen and CH3 from lipids and lipoproteins. The FLIm parameters included intensity ratios from channels (CH1/CH2, CH2/CH3, and CH1/CH3), average lifetime values, and the 12 Laguerre coefficient values from each of the channels CH1, CH2, and CH3. The analysis of raw FLIm data for the computation of the aforementioned FLIm parameters has been described in detail previously [12] and is briefly summarized in the supplemental material. The penetration of UV light in tissue is limited to 200–250 μm [13, 14]. As a result, FLIm parameters are not defined for deep lipid-rich core of a fibroatheroma (LFA).

### Statistical Analysis

FLIm-IVUS data from ROIs were fitted to a linear mixed effects model [15] to determine statistically significant correlations between various imaging parameters and pathological features. Statistically significant imaging parameters (*p* < 0.05) were used to train a support vector machine (SVM) classifier between different pathological features. A leave-one-out cross-validation test was performed to determine the sensitivity, specificity, and positive predictive value of FLIm, IVUS, and FLIm-IVUS parameters of detecting the eight pathological conditions over the entire data set. Details of the statistical analysis are summarized in the supplemental material.

## Results

Table 1 summarizes all the pathological features identified in this study along with the number of ROIs attributed to each feature from the 16 human LAD coronary specimens.

### FLIm-IVUS Data Visualization

**Fig. 1:**
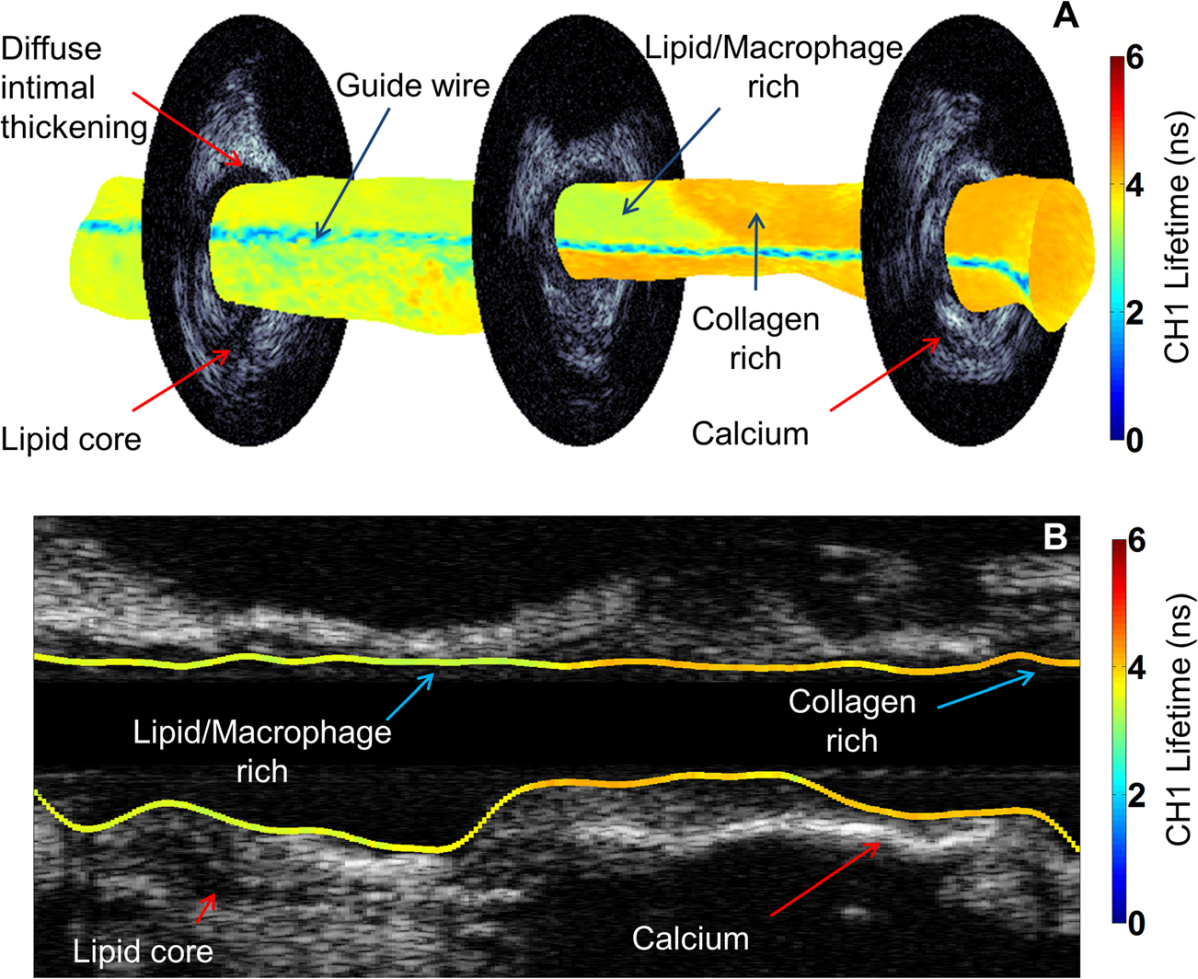
FLIm-IVUS data visualization. **a** CH1 fluorescence lifetime values mapped on to 3-D lumen surface. Three co-registered IVUS frames are shown. **b** Longitudinal IVUS image with CH1 fluorescence lifetime values mapped on to the lumen surface. Lower lifetime values indicate the presence of macrophages and lipid in plaque while higher lifetime values relate to increased collagen content. IVUS allows identification of calcium and lipid cores based on high and low echogenicities, respectively

**Fig. 2:**
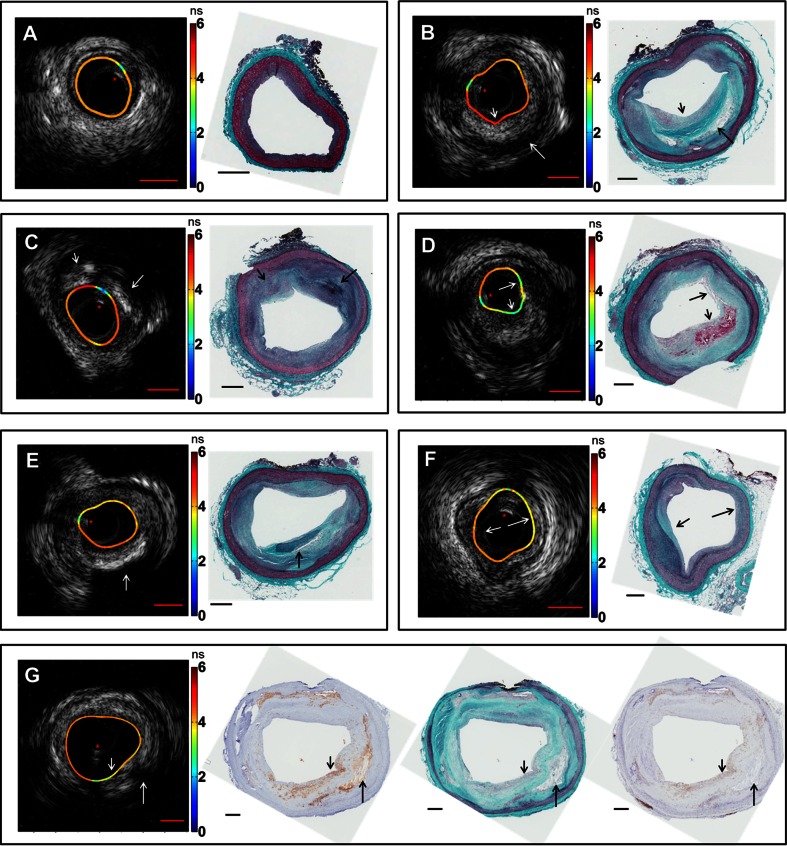
Representative images of co-registered IVUS, FLIm, and histology data showing different pathological features. Lifetime values (in ns) mapped onto the lumen surface are either from CH1 or CH2. Guide wire artifact is highlighted with a *red asterisk*. **a** DIT with uniform distribution of lifetime values over the lumen surface. IVUS shows minimal intimal thickening. **b** ThCFA and LFA. The thick cap (*small arrow*) is seen on both histology and IVUS with an underlying lipid-rich core (*large arrow*). Lifetime values at the cap region are higher due to increased amount of fibrous collagen. **c** PIT (*small arrow*) and FC (*large arrow*) are seen with varying echogenicities on IVUS as well as different lifetime values. **d** TCFA with intraplaque hemorrhage is seen with distinct lowering in lifetime values (*arrows*). IVUS is able to show burden of the TCFA core. **e** FC (*arrow*) is seen on IVUS as a region of high backscatter with a shadow. **f** FT is seen (*small arrow*) with increased lifetime values due to increase in collagen content as compared to regions with DIT (*large arrow*). **g** ThCFAM is seen with a fibrous cap (*small arrow*) rich in macrophages as confirmed in CD68-stained section (*middle*) along with CD45-stained section (*right*). Lipid-rich core (*large arrow*) is also seen on both IVUS and histology. Lowering in lifetime values is observed over the fibrous cap rich in macrophages compared to the surrounding regions in the lumen. Scale bars (*red*) on IVUS are 1 mm, and scale bars on histology (*black*) are 0.5 mm

**Fig. 3:**
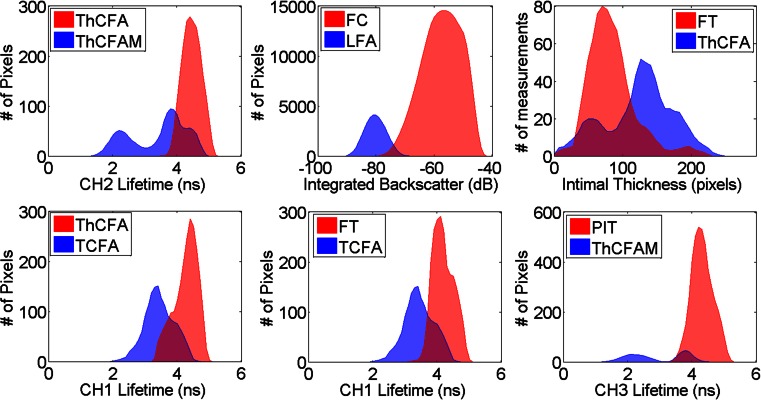
FLIm and IVUS parameter distribution for different pathology feature pairs. Parameters shown here are few of many that were found to be statistically different (post hoc Tukey’s test *p* < 0.001) between the pairs of pathological features (described in Table 1)

**Fig. 4:**
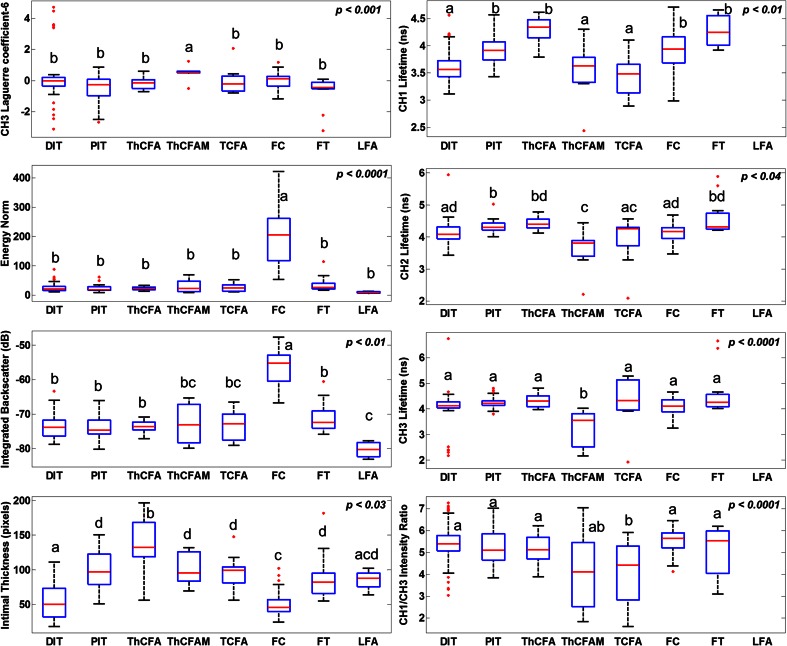
Box-whisker plots of select FLIm and IVUS parameters across all pathological features (described in Table 1). The *red bars* represent the median value, the *edges of the blue box* are the 25th and 75th percentiles, the *whiskers* extend to 1.5 times the *blue box* height, and outliers are plotted as *red dots*. Each box is labeled with one or more *letters* (*a*, *b*, *c*, or *d*). Boxes sharing no common letter are statistically different from one another (post hoc Tukey’s test *p*-value printed on *upper right corner* of each plot). Numerous other parameters were found to be able to discriminate between different pathological features; however, only a few parameters are shown here. No FLIm data are available for LFA due to limited penetration of UV light in tissue (200–250 μm). These parameters along with others (not shown) allow discriminating between any given pair of pathological features

An example of three-dimensional (3-D) visualization of co-registered FLIm (CH1 data) and IVUS data from a coronary specimen highlighting different morphological and biochemical features is shown in Fig. 1 (Video1.avi in the supplemental material). Such representation of the arterial vessel can aid the physician in the detection of different plaque types by allowing simultaneous visualization of such complimentary features in a clinical setting. Changes in fluorescence lifetime values have been attributed to changes in collagen, lipid, and macrophage content [6, 7] and are confirmed in images shown in Fig. 2 and data summarized in Figs. 3 and 4. This information, coupled with morphological features from IVUS such as IT, hyperechoic calcified tissue, and hypoechoic lipid cores, allows identification of relevant pathology described in Table 1.

### FLIm-IVUS Data and Pathology

Co-registered FLIm-IVUS images and histopathology data depicting different pathological features are shown in Fig. 2. Figures 3 and 4 summarize distributions of select statistically significant imaging parameters in relation to the pathological features. Data in this section are reported as mean ± SD along with confidence intervals at 99 % confidence level. Figure 2a shows a case with diffuse intimal thickening (DIT). Fluorescence lifetime values are seen to be uniform (CH1 lifetime for entire data set for DIT, 3.71 ± 0.34 ns (3.71–3.72 ns)) over the entire lumen surface with IVUS showing narrow to no intimal thickening. Figure 2b shows a case with ThCFA and LFA. A thick fibrous cap is seen in IVUS (small arrow), and lifetime values in this region are higher (CH1 lifetime for entire data set for ThCFA, 4.27 ± 0.33 ns (4.25–4.28 ns)) due to increase in collagen content as seen in histopathology. LFA (large arrow) is seen on IVUS as hypoechoic region (IB for entire data set for LFA, −80.2 ± 3.4 dB (−80.3–−80.2 dB)). Figure 2c shows a case with pathological intimal thickening (PIT, small arrow) and fibrocalcific plaque (FC, large arrow). PIT is seen with larger intimal thickening in IVUS and has higher average fluorescence lifetime (CH2 lifetime for entire data set for PIT, 4.28 ± 0.19 ns (4.28–4.30 ns)) compared to DIT (Fig. 4). FC is seen as hyperechoic region in IVUS (IB for entire data set for FC, −57.5 ± 6.6 dB (−57.6–−57.5 dB)) with loss of signal and shadowing behind the area of interest. The amount of fibrous tissue present in FC can be correlated to an increase in overall lifetime similar to PIT. However, FLIm alone is not able to distinguish between FC and fibrotic tissue (FT) due to similar collagen content, but the two features can be differentiated using IVUS (Fig. 4). Figure 2d shows a case with TCFA (both arrows) with intraplaque hemorrhage (large arrow). The spatial resolution of IVUS does not allow visualization of the thin cap. However, there is a significant decrease in fluorescence lifetime values in both regions (CH1 lifetime for entire data set for TCFA, 3.44 ± 0.44 ns (3.41–3.47 ns)) due to low collagen content and high lipid and macrophage content (CD68 stained pathology not shown). Intraplaque hemorrhage is often observed as low-echoic region in IVUS and may be confused with LFA. Figure 2e shows a case with FC which correlates to high backscatter signal in IVUS (arrow). CH1 lifetime values are higher than those observed in DIT (CH1 lifetime for entire data set for FC, 3.98 ± 0.39 ns (3.96–3.99 ns)). Figure 2f shows a case with FT and DIT where the region with FT has higher average lifetime values (CH1 lifetime for entire data set for FT, 4.19 ± 0.29 ns (4.17–4.20 ns)) than DIT. IVUS can distinguish between DIT and FT based on IT but not backscatter data alone (Fig. 4). Figure 2g shows a case with ThCFAM and LFA. Lowering of lifetime values is seen in the region infiltrated by macrophages (small arrow) as confirmed on a CD68-stained histopathology section (CH1 lifetime for entire data set for ThCFAM, 3.49 ± 0.71 ns (3.44–3.55 ns)). IVUS is not able to detect the presence of macrophages in fibrous caps but allows visualization of the plaque burden underneath (large arrow). Distributions for select FLIm and IVUS imaging parameters (post hoc Tukey’s test *p* < 0.001, as shown in Fig. 3) illustrate the ability of FLIm to distinguish between ThCFA and TCFA and detecting the presence of macrophages in fibrous caps (ThCFA vs. ThCFAM). These results (Figs. 3 and 4) emphasize that IVUS and FLIm together allow distinguishing between fibrolipidic plaques (TCFA, ThCFA, ThCFAM) from stable fibrous plaques (PIT, FT). Distribution of different imaging parameters across all pathological features can be appreciated from Fig. 4. A total of 47 imaging parameters (5 from IVUS and 42 from FLIm) were investigated for statistical significance. Of these, 39 were found to be able to distinguish between at least a pair of pathological features if not more. We show a select set of parameters (Figs. 3 and 4) that show the ability of FLIm and IVUS to distinguish between all the different pathological features. However, all the 39 statistically significant parameters were employed to train an SVM classifier.

### Classification Results

**Fig. 5:**
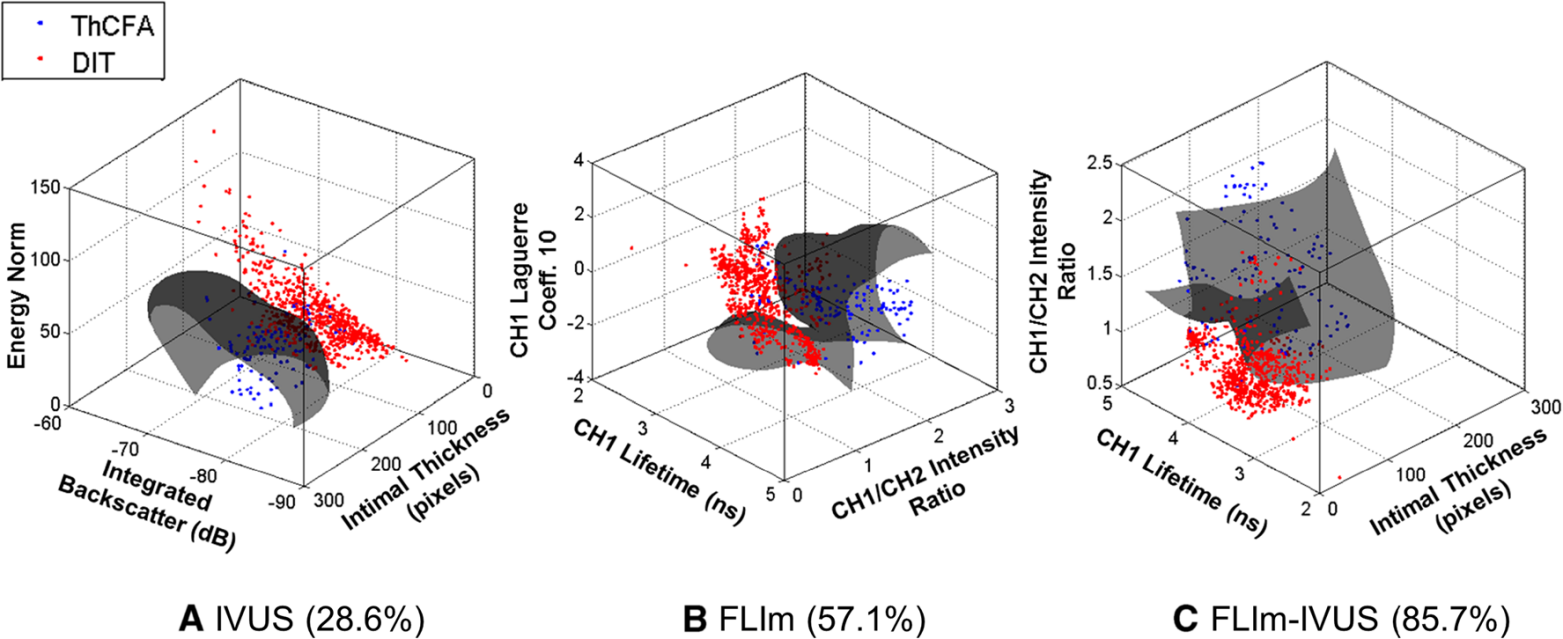
Three-dimensional scatter plots of training data for ThCFA and DIT using parameters from **a** IVUS, **b** FLIm, and **c** both FLIm and IVUS. Three imaging parameters were used for plotting purposes although up to 39 were used for the overall classification study. The SVM classifier hyperplane (*gray*) acts as a decision boundary for classification purposes. ThCFA detection sensitivity (reported in parenthesis) improves when both FLIm and IVUS parameters are used, enforcing the benefit of a bimodal imaging technique

Classification results are summarized in Table 2. A total of 184 ROIs attributed to different pathological features were used in the analyses. Thirty-nine of the 42 imaging parameters that were statistically significant (mixed effects ANOVA, *p* < 0.05, post hoc Tukey’s test, *p* < 0.05) were used to train SVM classifiers from FLIm, IVUS, and FLIm-IVUS data, respectively. Figure 5 shows 3-D scatter plots showing the data and the SVM classifier hyperplane for two features: ThCFA and DIT. The plots show an SVM built using three parameters; however, the overall classification was performed using SVMs built with all the statistically significant parameters. The plots illustrate the improvement in sensitivity when a classifier is trained using both IVUS and FLIm parameters as opposed to one or the other alone.

**Table 2:** Classification results summary

Feature	IVUS			FLIm			FLIm-IVUS		
	SN (%)	SP (%)	PPV (%)	SN (%)	SP (%)	PPV (%)	SN (%)	SP (%)	PPV (%)
DIT	81	81	71	100	96	93	87	100	100
PIT	50	84	40	94	98	91	84	100	100
ThCFA	29	99	80	57	100	100	86	100	100
ThCFAM	0	100	NaN	71	100	100	86	100	100
TCFA	0	100	NaN	90	100	100	80	100	100
FC	100	99	97	44	99	94	100	99	97
FT	0	100	NaN	100	90	40	92	99	92
LFA	100	91	19	0.0	100.0	NaN	100	92	21
Mean	45	94	61	70	98	88	89	99	89

*DIT* diffuse intimal thickening, *PIT* pathological intimal thickening, *ThCFA* thick-cap fibroatheroma, *ThCFAM* thick-cap fibroatheroma with macrophage/lymphocyte infiltration in cap, *TCFA* thin-cap fibroatheroma, *FC* fibrocalcific plaque, *FT* fibrotic tissue, *LFA* lipid-rich core of fibroatheroma, *SN* sensitivity, *SP* specificity, *PPV* positive predictive value, *IVUS* intravascular ultrasound, *FLIm* fluorescence lifetime imaging, *NaN* not a number (corresponds to division by zero)

IVUS imaging allowed reliable detection of FC and LFA as compared to FLIm alone. FLIm allowed detection of ThCFA, ThCFAM, and TCFA as observed from parameter distributions in Figs. 3 and 4. The sensitivity of detecting ThCFA and ThCFAM improved when both FLIm and IVUS data are used as compared to FLIm alone due to IT information available from IVUS. The classifier trained using FLIm parameters does not include the feature LFA, as no FLIm data are available for this feature due to limited penetration of UV light in tissue. As a result, the FLIm classifier appears to have better performance than the FLIm-IVUS classifier for certain features.

## Discussion

This study demonstrates for the first time that FLIm, a label-free spectroscopic rotational intravascular imaging technique, can complement morphological information from conventional IVUS with compositional information from the sub-intimal surface. FLIm-IVUS results from human LAD coronary arteries show the ability of this bimodal approach to detect different pathological features (Table 1) that are a subset of the modified classification scheme of Virmani et al. for atherosclerotic lesions [9]. Specifically, results (Figs. 3 and 4) show that FLIm allows differentiating between luminal areas histopathologically classified as TCFA from those classified as ThCFA as well as identifying macrophage infiltration in fibrous caps (ThCFAM). The clinical importance of detecting these plaque sub-types has been well emphasized [16]. Moreover, FLIm-IVUS data can be visualized concurrently (Figs. 1 and 2 and Video1.avi in the supplemental material) to comprehend both luminal composition and morphology of vessels simultaneously for enhanced plaque characterization that may serve as an invaluable tool to study plaque progression.

Compositional changes in tissue can be tracked by quantifying the autofluorescence of tissue components [17]. In this work, FLIm quantifies changes in fluorescence decay dynamics (e.g., average lifetime values and Laguerre coefficients) as a function of changes in arterial tissue composition (e.g., collagen, elastin, and lipids including cholesteryl oleate in macrophages). Thus, the accumulation of macrophages in the fibrotic cap and the co-localization of lipids change the overall fluorescence signature measured from tissue. While these compositional changes can only be assessed within 200–250 μm deep into the plaque due to limited penetration of UV light in tissue [13, 14], they provide valuable information about features in the diseased intima that have been associated with plaque instability and rupture. More importantly, for a plaque with a thick cap (ThCFA), FLIm will only be able to gather information from the cap and not from the underlying core. However, for caps that are relatively thin, such as in a TCFA (<65 μm), FLIm data will include information from both cap and part of the underlying core. Hence, despite the limited penetration depth of UV light, the ability of FLIm to resolve compositional changes in tissue makes it possible to identify TCFA from other plaque sub-types and detect macrophage infiltration (ThCFAM)—features that cannot be detected by IVUS alone.

In general, CH1 lifetime values are lowered due to the presence of macrophage and lipids, as well as due to reduction in collagen content as observed in data shown in Fig. 4. CH2 lifetime values can differentiate between ThCFA and TCFA while CH3 lifetime values can differentiate between ThCFAM and ThCFA (Fig. 4). IVUS provides morphological information such as intimal thickening, plaque burden, and presence of calcification that complements FLIm data for enhanced detection of different histologic subtypes in diseased coronary arteries. Besides possibly detecting vulnerable plaques, FLIm-IVUS imaging enables distinguishing between fibrous stiff plaques (FC, FT, and PIT) and lipid-rich plaques (TCFA, ThCFA, and ThCFAM) that may be important during PCI.

Qualitative results shown in Fig. 2 also illustrate the combined value of FLIm-IVUS imaging. Different plaque subtypes that cannot be distinguished by IVUS or FLIm alone are discerned by FLIm-IVUS synergism. SVM-based classification results also underscore the benefit of bimodal FLIm-IVUS arterial vessel evaluation (Table 2 and Fig. 5).

It is arguable that all eight different pathological features included in this study may not be relevant to a physician in a clinical setting. However, this study aims to highlight the sensitivity of our technique in relation to pathology that may be important for our ongoing research and studying plaque pathology.

### Study Limitations

Current configuration of the intravascular catheter and system requires that the FLIm-IVUS data be gathered sequentially rather than simultaneously. Thus, data acquisition and processing is rather long (~6 min). The use of graphics processing units to perform the same task can significantly reduce this time to allow real-time analysis (<1 min) in our future system. Moreover, our new catheter under development enables simultaneous FLIm-IVUS imaging by integrating the fiber optic and transducer on the same rotational axis. The registration error between FLIm and IVUS in the current study due to sequential imaging was found to be ~18° from phantom imaging experiments. As a result, ROIs defined by the pathologist that were less than 30° in span were not used in the study. However, this error will be avoided for future studies using the new catheter design.

Coronary segments in this study were imaged in saline and not in blood. In vivo imaging, however, will be performed by clearing the vessel of blood as is done during OCT imaging. Potential inadequate flushing or presence of blood may affect the FLIm spectroscopic findings which need to be quantified in future in vivo testing of this technique. Current work in progress is investigating flushing techniques to acquire FLIm-IVUS images in vivo in a pig using the new catheter system noted above. Flushing-related complications such as arrhythmia and hypotension that may arise will be addressed by developing a custom IVUS system to increase the pull-back speed and reduce flushing times. The limited penetration of UV light in tissue confines the ability of FLIm to interrogate only the biochemical composition of the luminal surface of the arterial wall. However, the compositional information gathered from this region is seen to be beneficial in complementing morphological data from IVUS in this study. Relative comparison of performances of various intravascular imaging techniques is shown in Table T1 in the supplemental material. The spatial resolution of FLIm used in this study is in the order of hundreds of microns (160 μm) but proves to be sufficient for imaging luminal surface features.

## Conclusions

This article reports the first results demonstrating the feasibility of a bimodal label-free tissue diagnostic technique combining rotational FLIm and IVUS for intravascular assessment of human coronary plaques. Current results demonstrate that FLIm technique can complement conventional IVUS and enable a better detection of various relevant pathological features that cannot be detected by IVUS alone. FLIm, through changes in tissue lifetime values, is capable of detecting the presence of macrophages in fibrous caps which is a prognostic factor for vulnerable plaques. It can also distinguish between TCFA and ThCFA amongst other pathological features. The changes in lifetime values are attributed to changes in lipid, macrophage, and collagen content in the intima. FLIm results coupled with morphological information from IVUS create a robust tool for staging plaques in arterial vessels. FLIm-IVUS bimodal data can be visualized in a clinical setting in 3-D or as cross-sectional images to aid the physician in the decision-making process during PCI. Results from this study lay the foundation for the future clinical applicability of FLIm-IVUS imaging to characterize plaques in vivo in real time, initially in a pig atherosclerotic model and, subsequently, in humans.

## Electronic supplementary material

Fig. S1 (GIF 23 kb)

High resolution image (TIFF 9777 kb)

Fig. S2 (GIF 27 kb)

High resolution image (TIFF 9793 kb)

Fig. S3 (GIF 4 kb)

High resolution image (TIFF 6675 kb)

ESM 1 (DOCX 135 kb)

Video 1 (AVI 19091 kb)

## Abbreviations

FLIm: Fluorescence lifetime imaging
IVUS: Intravascular ultrasound
PCI: Percutaneous coronary intervention
OCT: Optical coherence tomography
NIRS: Near-infrared reflectance spectroscopy
TCFA: Thin-cap fibroatheroma
ThCFA: Thick-cap fibroatheroma
LAD: Left anterior descending
UV: Ultraviolet
KHz: Kilohertz
MHz: Megahertz
ns: Nanosecond
rpm: Rotations per minute
nJ: Nanojoules
ROI: Region of interest
CD: Clusters of differentiation
UI: Ultrasound intensity
IB: Integrated backscatter
E: Energy norm
IT: Intimal thickness
R: Radial distance
CH1: Channel 1
CH2: Channel 2
CH3: Channel 3
LFA: Lipid-rich core of fibroatheroma
SVM: Support vector machine
DIT: Diffuse intimal thickening
PIT: Pathological intimal thickening
FC: Fibrocalcific plaque
FT: Fibrotic tissue
ThCFAM: Thick-cap fibroatheroma with macrophage/lymphocyte
ANOVA: Analysis of variance
