# Supplementary figures and images for: Fluorescence Lifetime Imaging Combined with Conventional Intravascular Ultrasound for Enhanced Assessment of Atherosclerotic Plaques: an Ex Vivo Study in Human Coronary Arteries
Source: J Cardiovasc Transl Res. 2015 May 1;8(4):253–63. doi: 10.1007/s12265-015-9627-3 (PMC4473095; doi:10.1007/s12265-015-9627-3)

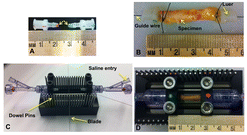

Supplement: Supplementary file 1 — (GIF 23 kb) [file 12265_2015_9627_Fig6_ESM.gif]

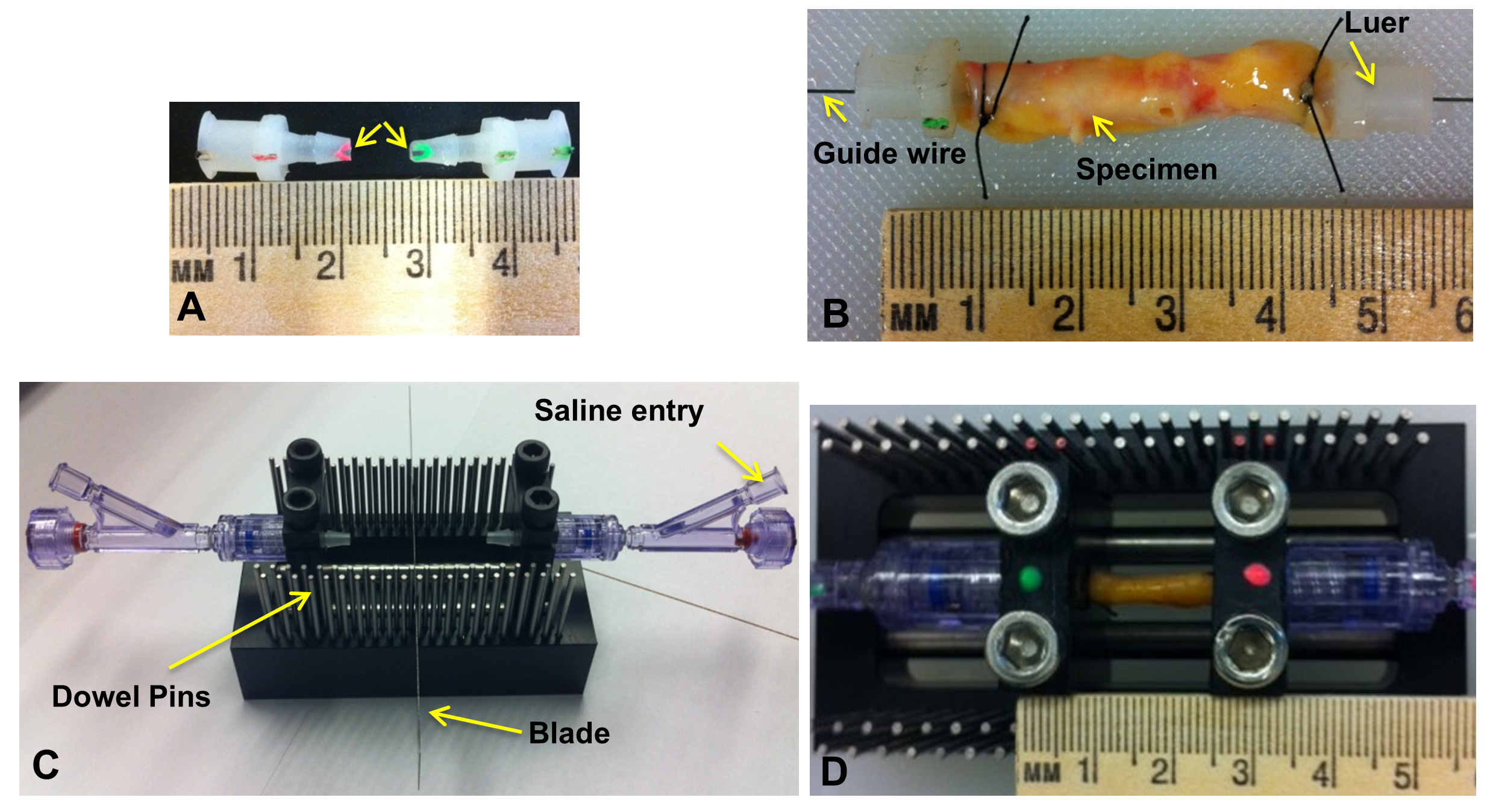

Supplement: Supplementary file 2 — High resolution image (TIFF 9777 kb) [file 12265_2015_9627_MOESM1_ESM.tif]

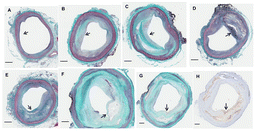

Supplement: Supplementary file 3 — (GIF 27 kb) [file 12265_2015_9627_Fig7_ESM.gif]

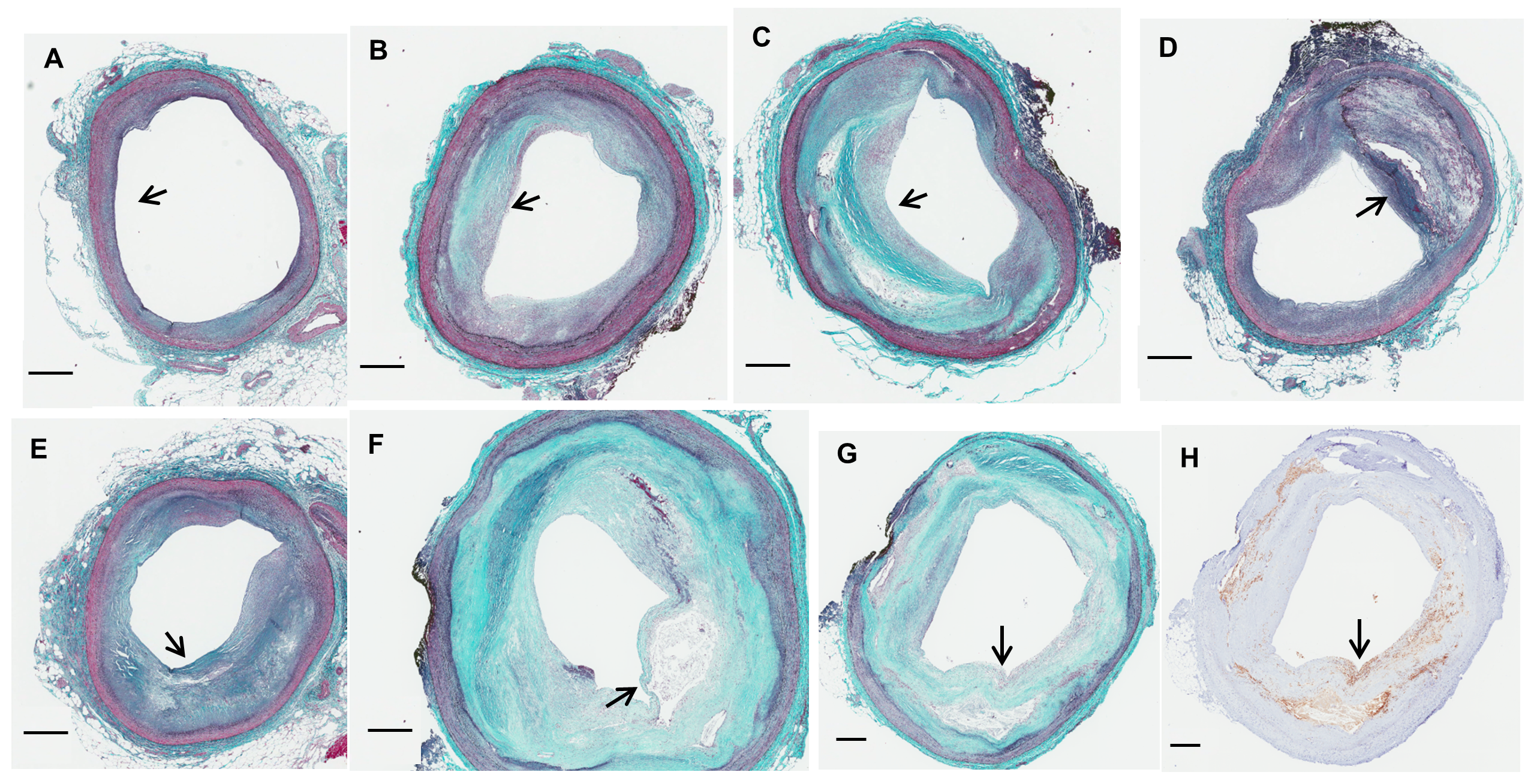

Supplement: Supplementary file 4 — High resolution image (TIFF 9793 kb) [file 12265_2015_9627_MOESM2_ESM.tif]

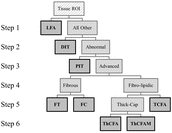

Supplement: Supplementary file 5 — (GIF 4 kb) [file 12265_2015_9627_Fig8_ESM.gif]

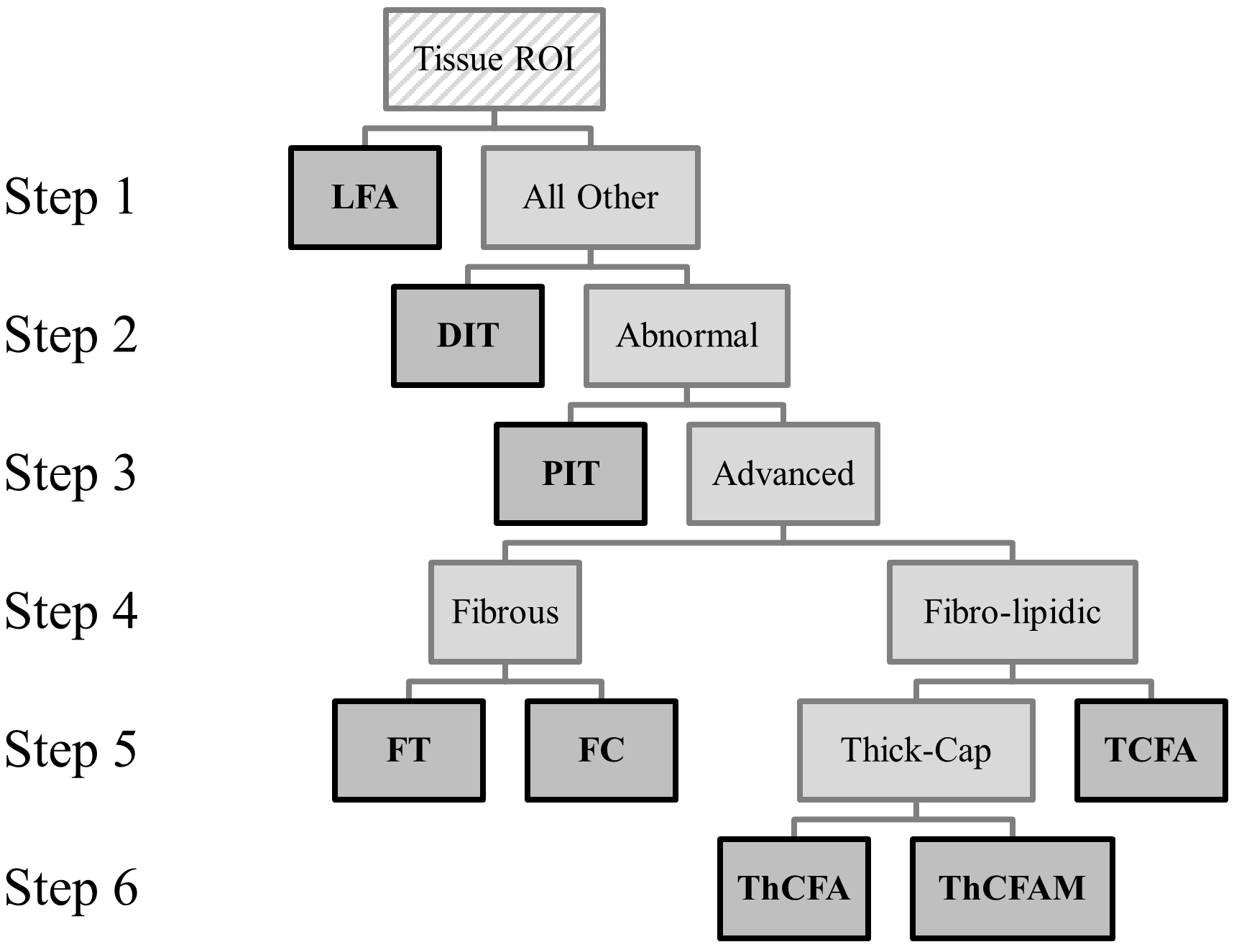

Supplement: Supplementary file 6 — High resolution image (TIFF 6675 kb) [file 12265_2015_9627_MOESM3_ESM.tif]
