# Supplementary material for: Fluorescence Lifetime Imaging Combined with Conventional Intravascular Ultrasound for Enhanced Assessment of Atherosclerotic Plaques: an Ex Vivo Study in Human Coronary Arteries
Source: J Cardiovasc Transl Res. 2015 May 1;8(4):253–63. doi: 10.1007/s12265-015-9627-3 (PMC4473095; doi:10.1007/s12265-015-9627-3)
Supplement: Supplementary file 7 — (DOCX 135 kb) [file 12265_2015_9627_MOESM4_ESM.docx]

# **ONLINE SUPPLEMENTAL MATERIAL**

**Fluorescence Lifetime Imaging Combined with Conventional Intravascular Ultrasound for Enhanced Assessment of Atherosclerotic Plaques: an Ex Vivo Study in Human Coronary Arteries**

Lead author’s first and last name: Hussain Fatakdawala

Short Title: FLIm-IVUS imaging of human coronaries

Hussain Fatakdawala^1^, BS; Dimitris Gorpas^1^, PhD; John W. Bishop^2^, MD; Julien Bec^1^, MS; Dinglong Ma^1^, BS; Jeffrey A. Southard^3^, MD; Kenneth B. Margulies^4^, MD; and Laura Marcu^1^, PhD.

^1^Department of Biomedical Engineering, University of California Davis , ^2^ Department of Pathology & Laboratory Medicine, University of California Davis Medical Center, ^3^Division of Cardiovascular Medicine, University of California Davis Medical Center,^4^ Cardiovascular Institute, Perelman School of Medicine, University of Pennsylvania.

Corresponding author:

Laura Marcu

University of California, Davis

451 E. Health Sciences Drive

Davis, CA, 95616

Tel: 530-752-0288

Email: [lmarcu@ucdavis.edu](mailto:lmarcu@ucdavis.edu)

# **Expanded Methods and Results**

# **Additional specimen details**

The arteries were obtained at the time of heart transplantation in 10 cases and at the time of organ procurement from brain-dead donors in 6 cases. Prospective informed consent for research use of explanted tissue was obtained for transplant recipients at the Hospital of the University Pennsylvania, and similar prospective consent for research use of non-failing hearts was obtained via the donor’s next-of-kin. Myocardial perfusion with ice-cold cardioplegia and prompt transport to the laboratory was performed as previously described [1]. The average age of patients providing tissues for these studies was 59±13 years (mean±SD). Thirteen of the patients were male and three were female. Among the 16 cases, five patients had a history of diabetes mellitus and six had a history of hypertension. All of the hearts from patients with advanced heart failure had a history of severe coronary artery disease, and five of them had prior coronary artery bypass grafting. In general, the degree of coronary artery disease was less severe among the hearts obtained from organ donors. The average length of the imaged samples was 10.1±3.3 mm (mean±SD) and the total imaged length from all samples was 161.6 mm.

# **FLIm Subsystem**

The fluorescence emission was collected using the same excitation fiber and was spectrally resolved in to three wavelength subbands (channels) using a series of 45^o^ dichroic beam splitters and band-pass filters (Semrock, Rochester, NY). The fluorescence subbands have a central wavelength/bandwidth of 390/40 nm (channel 1, CH1), 452/45 nm (channel 2, CH2) and 542/50 nm (channel 3, CH3) respectively. The signal was then coupled into three fibers with varying lengths of 1, 10 and 19 m to introduce time delays between the sub-band signals. The proximal ends of the fibers were coupled to a multichannel plate photomultiplier (MCP-PMT) with a 180 ps rise time (R5916U-50, Hamamatsu, Bridgewater, NJ). The electronic signals from the MCP-PMT are amplified by a preamplifier (1.5 GHz bandwidth, Hamamatsu, Japan) and recorded by a digitizer (National Instruments, PXIe 5185, 3 GHz bandwidth, 12.5 GHz sampling rate).

# **Specimen handling**

In order to perform FLIm-IVUS imaging, the specimen was thawed and rinsed with phosphate buffered saline (37^o^C) and fastened over two luers using surgical suture (Fig. S1A and S1B). A guide wire (0.014 inch diameter) was inserted into the lumen. Both luers presented a notch painted with fluorescent ink so that they were visible on both FLIm and IVUS images to allow axial and angular co-registration of both modalities. Additionally, the guide wire was also visible on both modalities to aid in angular registration. The specimen was then mounted into a custom built holder (Fig. S1C and S1D) with sliders to adjust for varying artery lengths. Additional connectors were attached to the distal end of the luers to allow catheter insertion and flushing with saline. The entire holder was immersed in a phosphate buffered saline bath maintained at 37^o^C during FLIm-IVUS imaging.

# **Tissue processing for co-registration of histology with imaging results**

After imaging was concluded, the top of the specimen in the holder was ablated linearly along the entire length of the specimen to create a histological marker. This marker was observed on each histological section marking the 12’oclock position during imaging. This ablation marker was aligned with the fluorescent markers on the luer for angular registration of histology with imaging data. The entire holder with the specimen was immersed in 10 % buffered formalin for tissue fixation for 24-72 hours. Post fixation the specimen was placed into decalcification solution for approximately 16 hours. The steel dowel pins on the sides of the holder helped guide a blade to allow precise cutting of tissue at 2 mm steps for axial registration between histology and imaging data. Cut samples were then sectioned (4 µm thick) and stained with hematoxylin and eosin, elastin, and Masson’s trichrome to visualize plaque morphology and composition (collagen, elastin, and lipid). Additionally, immunohistostain CD68 and CD45 was used to visualize macrophage and lymphocyte content in plaque respectively. A total of 87 histological sections were cut at 2 mm intervals from all specimens. For each cut, FLIm-IVUS data was averaged from the corresponding registered frame and the two adjacent frames.

# **Imaging Parameters**

# **FLIm data processing**

The measured fluorescence decay intensity $F(t)$at each point on the tissue ROI is a convolution of the actual fluorescence decay $p(t)$ and the instrument impulse response function$x(t)$. In discrete time, for $N$equal sampling time points$t_{i}=i\delta t$, $i=0.....,N-1$and sampling interval $\delta t$gives,

|  | $F(k)=\sum_{i=0}^{k} x(k-i)\cdot p(i),$ |  |
| --- | --- | --- |

where $k=0,\ldots,N-1$. $p(k)$ was expanded on to a set of discrete ordered Laguerre basis functions, $b_{l}$ such that,

|  | $p(k)=\sum_{l=0}^{L-1} c_{l}b_{l}(k;\alpha),$ |  |
| --- | --- | --- |

where $L$and$\alpha$ are the basis parameters and $c_{l}$ is the $l^{th}$expansion coefficient. Using this formulation $p(k)$was computed using a fast constrained least square deconvolution technique [2]. The average lifetime was calculated from $p(k)$ as follows,

|  | $\tau_{avg}=\frac{\delta t\sum_{k} kp(k)}{\sum_{k} p(k)}.$ |  |
| --- | --- | --- |

Consequently, for every measurement in a given ROI, the absolute fluorescence intensity, fluorescence lifetime and Laguerre expansion coefficients are computed to generate 2-D intensity, average lifetime and Laguerre coefficient maps. The fluorescence intensity is computed as the numerical integration of the decay curve $p(k)$ for each wavelength sub-band. A 12 order Laguerre expansion was used in this study and hence a total of 36 Laguerre coefficients from three different channels are reported.

# **IVUS data processing**

IVUS radio frequency (RF) data were used to reconstruct a 2-D image by displaying the log compressed amplitude or ultrasound intensity (UI). The amplitude is derived from the Hilbert transform of the scanning A-line data. DC offset was subtracted from the data and a band pass filter (25-60 MHz) was employed for noise reduction.

IVUS raw RF data were also used to compute the backscatter coefficient function$B_{c}(f)$, integrated backscatter (IB) values and the energy norm (E). These served as quantitative IVUS parameters for studying IVUS data in relation to different pathology.

$B_{c}(f)$ for a given ROI was computed by correcting for the system response by normalizing the tissue ROI spectra with the reference spectra:

|  | $B_{c}(f)=\frac{H_{S}(z,f)}{H_{R}(z,f)}$ |  |
| --- | --- | --- |

where $H_{S}(z,f)$ and $H_{R}(z,f)$are the frequency $f$and depth (z) dependent spectra obtained from the ultrasound signal from the tissue ROI and a reflector-liquid interface respectively. Both spectra are estimated by calculating the fast Fourier transform (FFT) of the Hamming windowed segments of the corresponding backscattered signal. IB value was computed from the backscatter coefficient function in dB, $B_{c}^{dB}(f)$ (logarithm of the spatially averaged$B_{c}(f)$) over the bandwidth $\Delta f$of$H_{R}(z,f)$

|  | $B_{c}^{dB}=10\log_{10}\left\langle\left\vert B_{c}(f)\right\vert^{2}\right\rangle$ |  |
| --- | --- | --- |

|  | $IB=\frac{1}{\Delta f}\int_{\Delta f} B_{c}^{dB}(f)df$ |  |
| --- | --- | --- |

The energy norm (E) of a given region of interest is defined as:

|  | $E=\sqrt{w.w^{T}}$ |  |
| --- | --- | --- |

where $w$is the Hamming windowed segments of the corresponding backscattered RF signal. The energy norm depicts the amount of absolute ultrasound energy backscattered from a sample.

Additionally, the intimal thickness (IT) and the radial distance from the transducer (R) for every ROI were also estimated.

# **Statistics**

FLIm-IVUS data from ROIs were fitted to a linear mixed effects model [3] to determine statistically significant correlations between various imaging parameters and pathological features. The statistical significance of each imaging parameter over the entire data set was determined by performing an analysis of variance (ANOVA) followed by a false discovery rate correction (Benjamini Hochberg test, p<0.05). Finally, for every significant imaging parameter a post-hoc Tukey’s test was performed to determine p-values for every possible pair of pathological features. This allowed selecting a subset of imaging parameters (p < 0.05) from the total of 47 parameters (5 from IVUS and 42 from FLIm) to train a support vector machine (SVM) classifier between different pathological features. A leave-one-out cross-validation test was performed to determine the sensitivity, specificity and positive predictive value of FLIm, IVUS and FLIm-IVUS parameters of detecting the eight pathological conditions over the entire data set. All analyses were performed using statistical packages [4,5] in R and MATLAB.

Support vector machine (SVM) is a supervised learning model that allows analyzing and recognizing patterns in data for classification [6]. The basic idea of SVM is to map original binary data or observations (belonging to one of two groups) onto a new space defined by a kernel function. In this space, the data are linearly separable by an optimal line (2D data) or plane (high dimensional data). This plane is determined by maximizing the distance of the data points (known as support vectors) from this plane. The support vectors are a subset of the data at the interface of the two groups. When the plane is projected back to the original data space, it forms an irregular surface known as the hyperplane. This hyperplane serves as a decision boundary that is used to classify new observations into either group. An observation belongs to one or the other group depending on which side of the plane it falls on. In this study, we used the radial basis function as our kernel to train SVMs.

A hierarchical tree (Fig. S3) was designed to perform classification of tissue ROIs. Based on this tree, a total of seven SVMs were trained, one for each step shown in Fig. S3, except for step 5 which has two SVMs. For any given ROI, an SVM is used to classify it in to one of two groups starting at step 1 (LFA or All others). This process is continued (step 2 and beyond) until the ROI is attributed to one of the eight features (boxes with bolded font in Fig. S3).

Classification evaluation is performed using a leave-one-out cross-validation method where the entire data set except one observation (ROI) is used to train the seven SVMs. The left out observation is then classified following the hierarchical tree and using the trained SVMs. The process is repeated till every observation in the data set has been left out and classified. Results are used to compute the sensitivity, specificity and positive predictive value of the SVM.

# **Additional Tables**

Table T1. Comparison of performances between different intravascular imaging techniques

|  | IVUS | IVUS-VH | OCT | NIRS | FLIm |
| --- | --- | --- | --- | --- | --- |
| Imaging depth in tissue | +++ [7,8] | +++ [7,8] | ++ [8] | ++ [9] | + |
| Spatial resolution | ++ [10] | ++ [10] | +++ [8] | NA | NA |
| Resolve intima composition: |  |  |  |  |  |
| - Lipid | — [11] | ++ [12] | + [13] | +++ [14,15] | +++ [16] |
| - Collagen and elastin | — [11] | + [12] | + [13] | + [17] | +++ [16] |
| - Macrophages | — [11] | — [11] | ++ [18] | + [17] | +++ [16,19] |
| Image outward remodeling | +++ [20] | +++ [20] | — [21] | — | — |
| Detect lipid-cores | + [22] | ++ [12] | + [23] | ++ [14,15] | — |
| Require blood-free field of view | No | No | Yes | No | Yes |

IVUS indicates intravascular ultrasound; IVUS-VH, intravascular ultrasound – virtual histology; OCT, optical coherence tomography; NIRS, near infra-red spectroscopy; FLIm, fluorescence lifetime imaging. Methods’ feature or capability to perform is rated as unable (—), low performance (+), moderate performance (++), high performance (+++) and not applicable (NA).

# **Additional Figures Legends**

**Fig. S1**. (A) Luers with fluorescent ink painted notches (arrows) to allow axial registration between IVUS and FLIm images. (B) Specimen fastened on luers with a guide wire placed in the lumen. (C) Custom built holder with dowel pins to guide a blade for cutting tissue at 2 mm intervals. (D) Specimen fastened on luers mounted on to the holder for FLIm-IVUS imaging.

**Fig. S2**. Representative histology images depicting the different pathological conditions identified in this study. Elastin- Masson’s Trichrome stained section showing (arrows) (A) DIT, where the intima shows sparse thickening, (B) PIT, where the intima shows more profound intimal thickening causing narrowing of the lumen. The intima has high collagen content. (C) ThCFA with underlying LFA, where the intima shows the presence of a fibrous collagen rich cap with an underlying core rich in lipids, cholesterol and macrophages, (D) FC, where the intima shows presence of a large fibroatheroma predominant with collagen and calcium. (E) FT, where the intima shows presence of acellular fibrous dense collagen fibers. (F) TCFA, where the intima shows presence of a thin fibrous cap with an underlying core rich in lipids, cholesterol and macrophages. The thin cap also have macrophages, (G) ThCFAM, where the intima shows features similar to ThCFA except that the cap also has high infiltration of macrophages as confirmed in (H) showing a CD68 stained (hematoxylin counter stain) where macrophage infiltration (brown) is seen in fibrous cap and core. Sections shown in G and H are the same specimen. Scale bars (black) are 0.5 mm.

**Fig. S3.** Hierarchical tree used to perform classification of any given tissue ROI. At each step (1-6), an SVM is used to classify the ROI in to one of two groups. The process is concluded at a given step if the ROI is attributed to one of the eight features (bolded boxes). DIT indicates diffuse intimal thickening; PIT, pathological intimal thickening; ThCFA, thick cap fibroatheroma; ThCFAM, thick cap fibroatheroma with macrophage/lymphocyte infiltration in cap; TCFA, thin cap fibroatheroma; FC, fibro-calcific plaque; FT, fibrotic tissue; LFA, lipid rich core of fibroatheroma, “All Other” includes all features except LFA, “Abnormal” includes all features except LFA and DIT, “Advanced” includes all features except LFA, DIT and PIT, “Fibrous” includes FC and FT, “Fibrolipidic” includes all thick-cap features (ThCFA and ThCFAM) and TCFA.

**Video1**. The video shows 2-D and 3-D visualization of fly-through of FLIm-IVUS data from a human LAD coronary specimen. Such visualizations allow comprehending both morphological and compositional features of plaques that may serve as a valuable tool to a cardiologist. Future work entails development of a system to allow quasi-real time data processing to allow such visualization in a clinical setting.

**Supplemental References**

1. Dipla, K., Mattiello, J. A., Jeevanandam, V., Houser, S. R., & Margulies, K. B. (1998). Myocyte recovery after mechanical circulatory support in humans with end-stage heart failure. *Circulation, 97*(23), 2316-2322.

2. Liu, J., Sun, Y., Qi, J., & Marcu, L. (2012). A novel method for fast and robust estimation of fluorescence decay dynamics using constrained least-squares deconvolution with Laguerre expansion. *Physics in Medicine and Biology, 57*(4), 843-865, doi:10.1088/0031-9155/57/4/843.

3. Lindstrom, M. J., & Bates, D. M. (1988). Newton-Raphson and Em Algorithms for Linear Mixed-Effects Models for Repeated-Measures Data. *Journal of the American Statistical Association, 83*(404), 1014-1022.

4. Pinheiro, J., Bates, D., Debroy, S., Sarkar, D., & The R Development Core team (2013). *nlme: Linear and Nonlinear Mixed Effects Models*.

5. Hothorn, T., Bretz, F., & Westfall, P. (2008). Simultaneous inference in general parametric models. *Biom J, 50*(3), 346-363, doi:10.1002/bimj.200810425.

6. Cristianini, N., & Shawe-Taylor, J. (2000). *An introduction to support Vector Machines: and other kernel-based learning methods*: Cambridge University Press.

7. Elliott, M. R., & Thrush, A. J. (1996). Measurement of resolution in intravascular ultrasound images. *Physiol Meas, 17*(4), 259-265.

8. Brezinski, M. E., Tearney, G. J., Weissman, N. J., Boppart, S. A., Bouma, B. E., Hee, M. R., et al. (1997). Assessing atherosclerotic plaque morphology: comparison of optical coherence tomography and high frequency intravascular ultrasound. *Heart, 77*(5), 397-403.

9. Wang, J., Geng, Y.-J., Guo, B., Klima, T., Lal, B. N., Willerson, J. T., et al. (2002). Near-infrared spectroscopic characterization of human advanced atherosclerotic plaques. *Journal of the American College of Cardiology, 39*(8), 1305-1313, doi:10.1016/S0735-1097(02)01767-9.

10. Nissen, S. E., & Yock, P. (2001). Intravascular ultrasound: novel pathophysiological insights and current clinical applications. *Circulation, 103*(4), 604-616.

11. Suh, W. M., Seto, A. H., Margey, R. J., Cruz-Gonzalez, I., & Jang, I. K. (2011). Intravascular detection of the vulnerable plaque. *Circ Cardiovasc Imaging, 4*(2), 169-178, doi:10.1161/CIRCIMAGING.110.958777.

12. Nair, A., Kuban, B. D., Tuzcu, E. M., Schoenhagen, P., Nissen, S. E., & Vince, D. G. (2002). Coronary plaque classification with intravascular ultrasound radiofrequency data analysis. *Circulation, 106*(17), 2200-2206.

13. Tearney, G. J., Regar, E., Akasaka, T., Adriaenssens, T., Barlis, P., Bezerra, H. G., et al. (2012). Consensus standards for acquisition, measurement, and reporting of intravascular optical coherence tomography studies: a report from the International Working Group for Intravascular Optical Coherence Tomography Standardization and Validation. *J Am Coll Cardiol, 59*(12), 1058-1072, doi:10.1016/j.jacc.2011.09.079.

14. Gardner, C. M., Tan, H., Hull, E. L., Lisauskas, J. B., Sum, S. T., Meese, T. M., et al. (2008). Detection of Lipid Core Coronary Plaques in Autopsy Specimens With a Novel Catheter-Based Near-Infrared Spectroscopy System. *JACC: Cardiovascular Imaging, 1*(5), 638-648, doi:<http://dx.doi.org/10.1016/j.jcmg.2008.06.001>.

15. Waxman, S., Dixon, S. R., L'Allier, P., Moses, J. W., Petersen, J. L., Cutlip, D., et al. (2009). In Vivo Validation of a Catheter-Based Near-Infrared Spectroscopy System for Detection of Lipid Core Coronary Plaques: Initial Results of the SPECTACL Study. *JACC: Cardiovascular Imaging, 2*(7), 858-868, doi:<http://dx.doi.org/10.1016/j.jcmg.2009.05.001>.

16. Phipps, J., Sun, Y., Saroufeem, R., Hatami, N., Fishbein, M. C., & Marcu, L. (2011). Fluorescence lifetime imaging for the characterization of the biochemical composition of atherosclerotic plaques. *J Biomed Opt, 16*(9), 096018, doi:10.1117/1.3626865.

17. Moreno, P. R., Lodder, R. A., Purushothaman, K. R., Charash, W. E., O’Connor, W. N., & Muller, J. E. (2002). Detection of Lipid Pool, Thin Fibrous Cap, and Inflammatory Cells in Human Aortic Atherosclerotic Plaques by Near-Infrared Spectroscopy. *Circulation, 105*(8), 923-927, doi:10.1161/hc0802.104291.

18. Tearney, G. J., Yabushita, H., Houser, S. L., Aretz, H. T., Jang, I. K., Schlendorf, K. H., et al. (2003). Quantification of macrophage content in atherosclerotic plaques by optical coherence tomography. *Circulation, 107*(1), 113-119, doi:10.1161/01.cir.0000044384.41037.43.

19. Phipps, J. E., Hatami, N., Galis, Z. S., Baker, J. D., Fishbein, M. C., & Marcu, L. (2011). A fluorescence lifetime spectroscopy study of matrix metalloproteinases-2 and -9 in human atherosclerotic plaque. *J Biophotonics, 4*(9), 650-658, doi:10.1002/jbio.201100042.

20. Maehara, A., Mintz, G. S., Bui, A. B., Walter, O. R., Castagna, M. T., Canos, D., et al. (2002). Morphologic and angiographic features of coronary plaque rupture detected by intravascular ultrasound. *Journal of the American College of Cardiology, 40*(5), 904-910, doi:<http://dx.doi.org/10.1016/S0735-1097(02)02047-8>.

21. Maehara, A., Mintz, G. S., & Weissman, N. J. (2009). Advances in Intravascular Imaging. *Circulation: Cardiovascular Interventions, 2*(5), 482-490, doi:10.1161/circinterventions.109.868398.

22. Prati, E., Arbustini, E., Labellarte, A., Dal Bello, B., Mallus, M. T., Sommariva, L., et al. (2000). Intravascular ultrasound insights into plaque composition. *Zeitschrift Fur Kardiologie, 89*, 117-123.

23. Manfrini, O., Mont, E., Leone, O., Arbustini, E., Eusebi, V., Virmani, R., et al. (2006). Sources of Error and Interpretation of Plaque Morphology by Optical Coherence Tomography. *The American Journal of Cardiology, 98*(2), 156-159, doi:<http://dx.doi.org/10.1016/j.amjcard.2006.01.097>.
